# Supplementary figures and images for: TNF-α induces VE-cadherin-dependent gap/JAIL cycling through an intermediate state essential for neutrophil transmigration
Source: Front Immunol. 2025 Sep 9;16:1665264. doi: 10.3389/fimmu.2025.1665264 (PMC12454053; doi:10.3389/fimmu.2025.1665264)

# Supplementary Figure S1

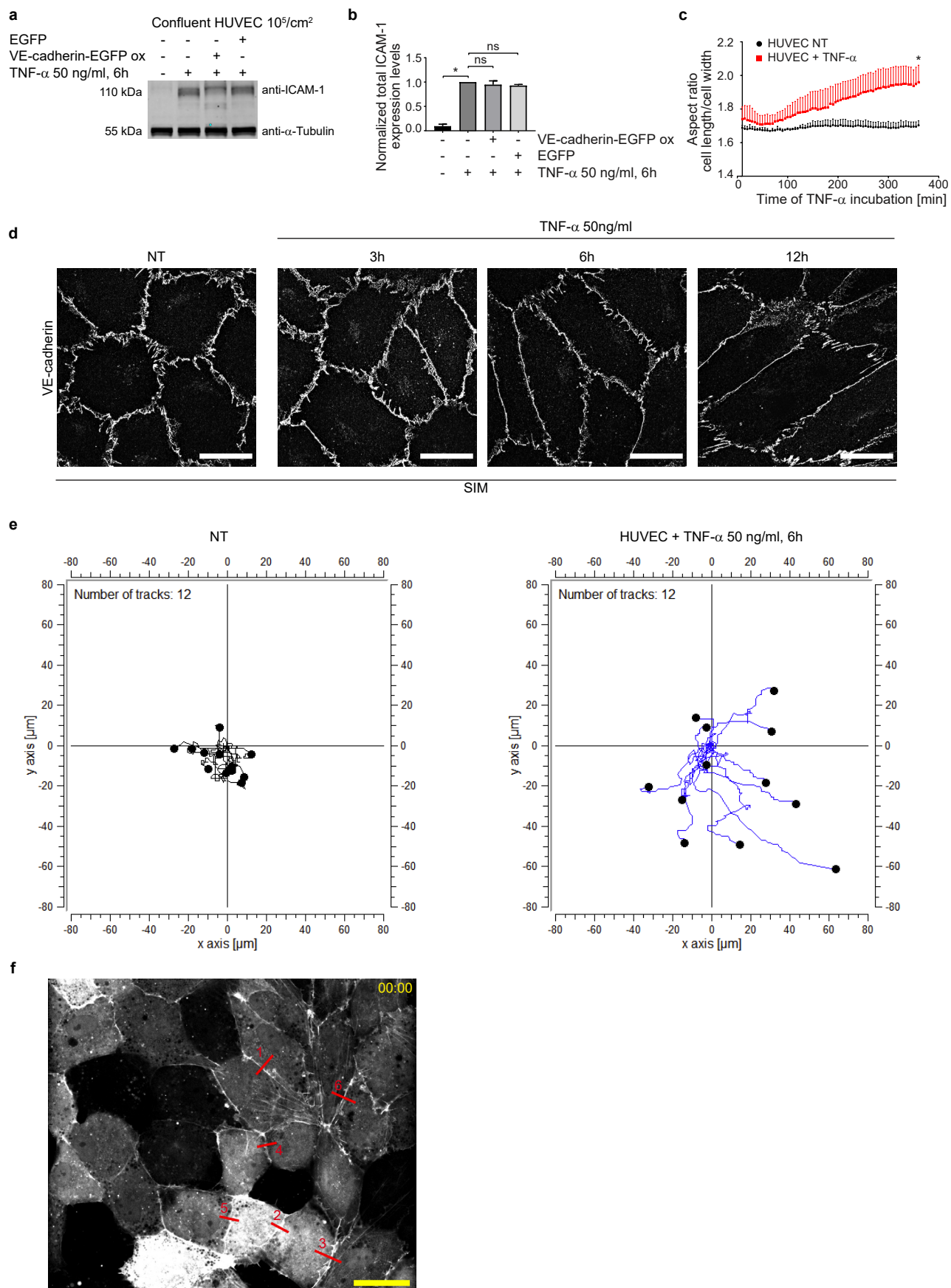

Supplement: Supplementary Figure 1 — Western blot analysis of ICAM-1 from naive, EGFP- and VE-cadherin-EGFP-overexpressing HUVEC after 6 hours of TNF-α treatment. (a) Western blot analyses of HUVEC treated as indicated. (b) Quantification of ICAM-1 expression levels (a, b): n=3 independent experiments; Kruskal-Wallis test). (c) Analysis of the cell aspect ratio upon TNF-α treatment; n=3 independent experiments, considering the mean values of 6 different locations at 10x magnification. Each position contains approximately 1800 +/- 200 cells per time point; unpaired t-test. (d) Shape change in HUVEC after 3h, 6h and 12h of TNF-α stimulation. Scale bar: 20 µm. (e) Cell migration plots of control HUVEC and after TNF-α stimulation. (f) Overview used for line scan analysis depicted in Figure 1 (g-i). Scale bar 30 µm. ns= not significant. [file DataSheet1.pdf]

Supplementary Figure S2

a

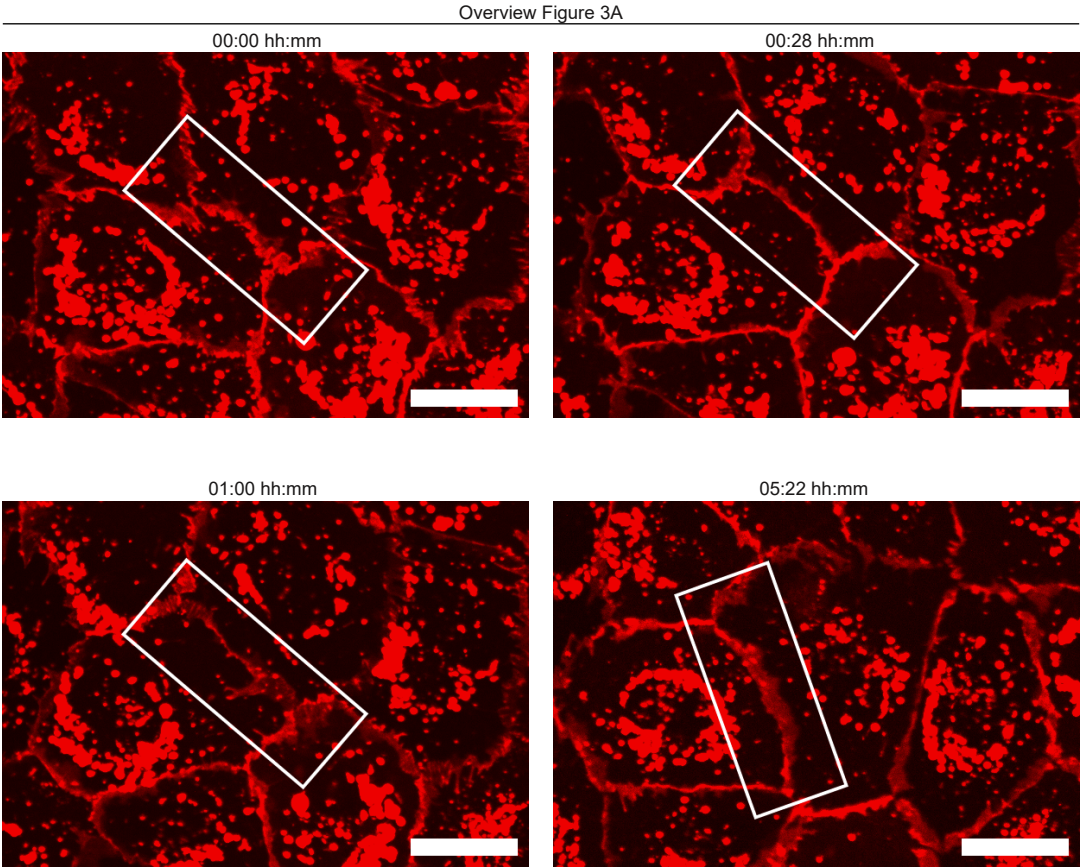

Supplement: Supplementary Figure 2 — Overview of Figure 2a . Scale bar: 20 µm. [file DataSheet2.pdf]

### Supplementary Figure S3

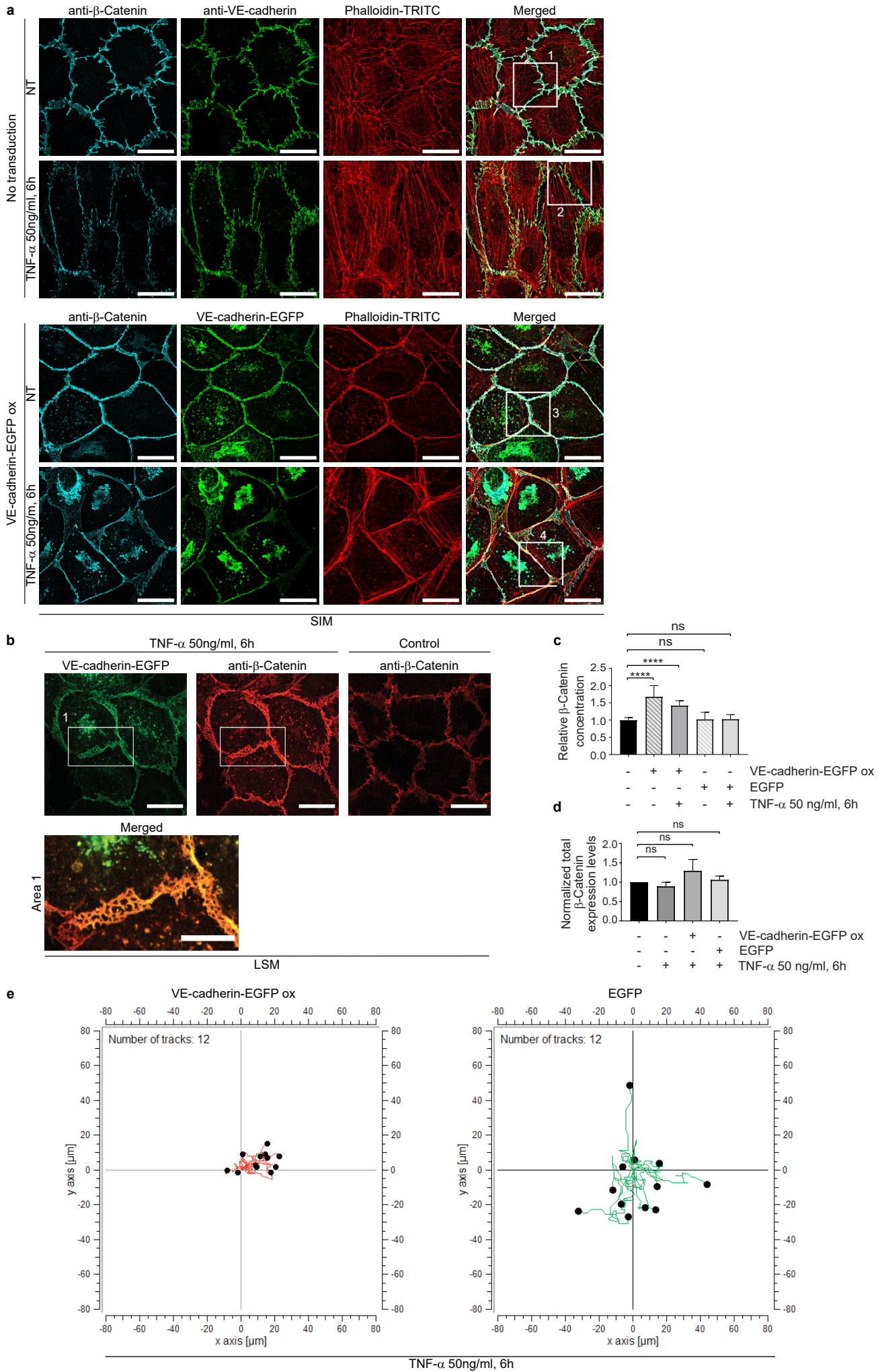

Supplement: Supplementary Figure 3 — (a) Overview of Figure 4a . (b) LSM-images of VE-cadherin and β-Catenin. Note: Orange color indicates VE-cadherin-EGFP and β-Catenin superimposition. (c) Quantification of the relative β-Catenin concentrations of LSM-images as depicted in b in transduced and untransduced cells following TNF-α treatment. N= 3 independent experiments with the following number of LSM images (63x magnification) included in total per condition. NT, n= 52; VE-cadherin-EGFP, n=17; VE-cadherin-EGFP+ TNF-α, n=33; EGFP, n=18; EGFP+ TNF-α, n= 33; ordinary one-way ANOVA. Scale bar overview, 20 µm; scale bar cropped areas, 10 µm. (d) Quantification of β-catenin expression levels, as analyzed by Western blot in Figure 4b . 4 WBs from n= 3 independent experiments; Ordinary one-way ANOVA. (e) Cell migration plots of VE-cadherin-EGFP and EGFP overexpressing HUVEC following TNF-α stimulation. 12 single cells per condition are shown. ns= not significant. [file DataSheet3.pdf]

# Supplementary Figure S4

**a**

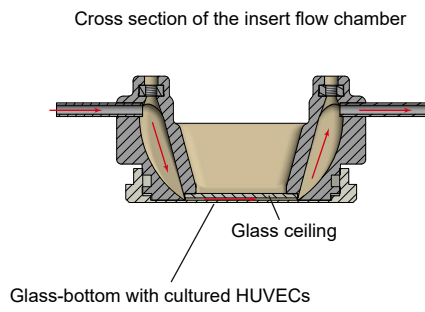

**b**

Overview Figure TEM 5c

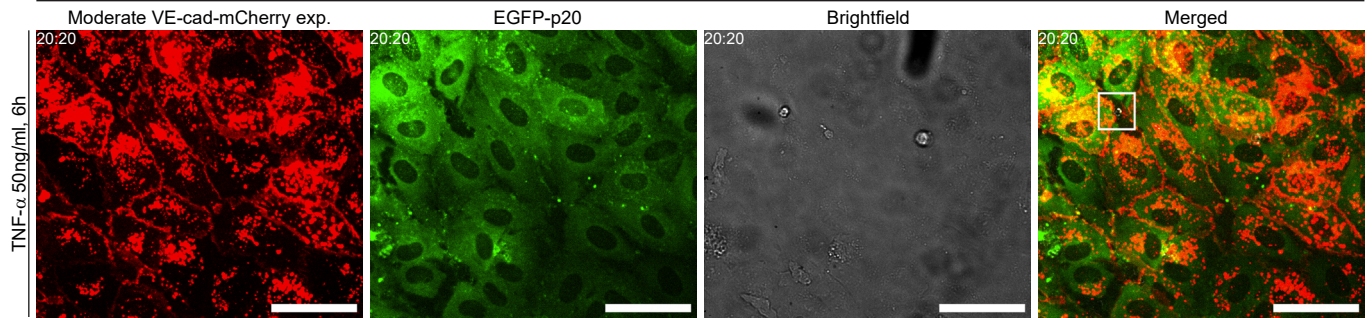

**c**

Overview Figure 5d

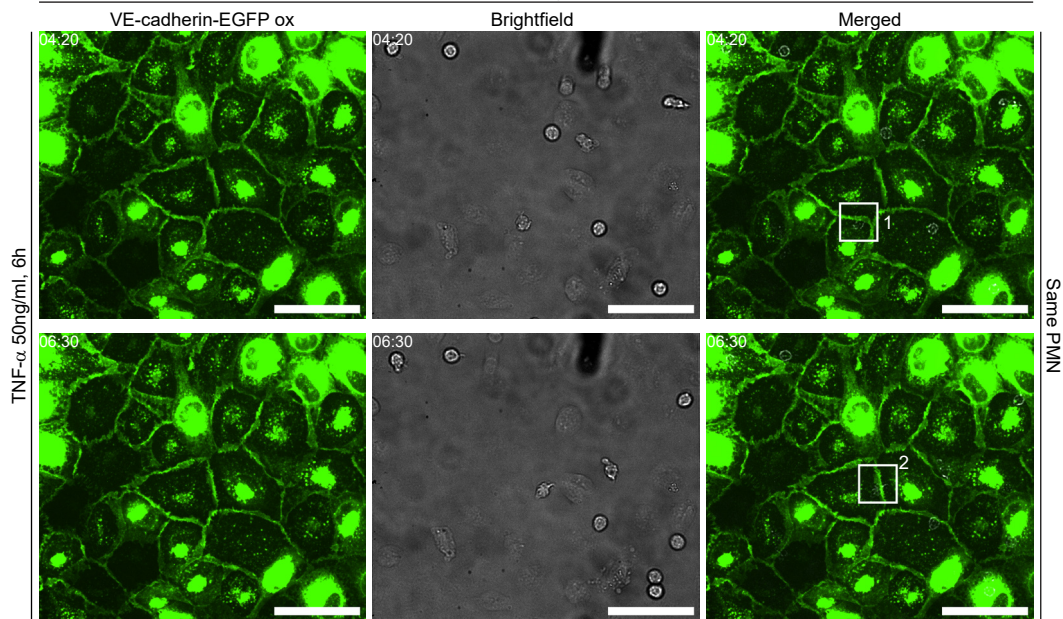

**d**

Overview Figure TEM 5e

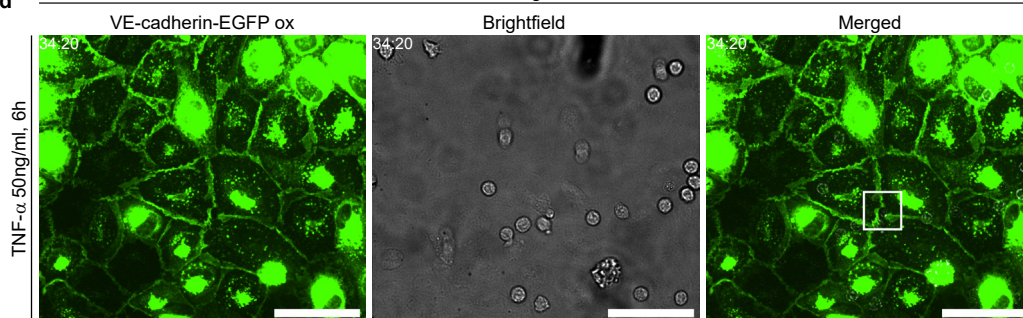

Supplement: Supplementary Figure 4 — (a) Cross section of the custom-made insert flow chamber. (b) Overview of TEM shown in Figure 5c . Scale bar: 20 µm. (c) Overview of two areas shown in Figure 5d . Scale bar: 20 µm. (d) Overview of TEM depicted in Figure 5e . Scale bar: 20 µm. [file DataSheet4.pdf]
